# An Updated Systematic Review of Topical Corticosteroid Withdrawal (TCSW): Steroid Addiction or Adverse Drug Effect?

**DOI:** 10.7759/cureus.107365

**Published:** 2026-04-19

**Authors:** Hunjung Choi, Maria Orbe, Sahla Esam, Jordan Parker

**Affiliations:** 1 Medical School, University of Missouri School of Medicine, Columbia, USA; 2 Dermatology, University of Missouri School of Medicine, Columbia, USA

**Keywords:** corticosteroid, rebound, rosacea, tolerance, topical corticosteroid withdrawal, withdrawal

## Abstract

Topical corticosteroid withdrawal (TCSW) can occur after prolonged topical corticosteroid (TCS) use. Despite the effectiveness of TCS in treating dermatological conditions, the phenomenon of steroid phobia has been gaining attention on social media platforms. This has contributed to increased anxiety among patients and potentially influenced their treatment adherence. This review aims to update the findings of two studies by investigating the latest research on TCSW. A systematic search was conducted in Ovid MEDLINE, PubMed, and the Cochrane Library from October 2020 to June 2024. Seven studies were included in the analysis, including five case reports, one case series, and one qualitative cross-sectional survey, reflecting the limited and descriptive nature of the currently available evidence on TCSW. TCSW appears to predominantly occur in adult women (90%) using TCS for atopic dermatitis (99.5%), mostly on the face (90%). Common symptoms include burning (89.8%), itching (85.6%), and skin hypersensitivity (82.3%), with erythema (89.6%) and desquamation (88.7%). TCSW appears to occur more commonly in women using high-potency TCS on the face long-term. Inconsistent diagnostic criteria, variable definitions, and the primarily descriptive nature of the currently available evidence complicate accurate diagnosis and treatment of TCSW. Increased awareness, education, and research on standardized criteria and management strategies are essential.

## Introduction and background

Topical corticosteroids (TCS) have been a cornerstone in the treatment of various dermatological conditions for many years, and their effectiveness is well-demonstrated [1]. Despite their long history of successful use, a phenomenon known as “steroid phobia” has recently gained attention, particularly on social media [2-4]. Steroid phobia is characterized by a fear of becoming dependent or addicted to topical steroids and concerns about never being able to discontinue their use due to potential withdrawal or rebound effects. With increasing attention to steroid phobia among the general public, patients’ engagement and adherence to TCS treatment are significantly impacted [2-4]. With the rising awareness of topical corticosteroid withdrawal (TCSW), the National Eczema Association recognized TCSW, or topical steroid withdrawal (TSW), as a serious potential side effect, noting that much is still unknown about the condition [5].

To address TCSW, Hajar et al. published a systematic review in 2015, analyzing the available evidence of TCSW in patients following chronic TCS use [6]. Subsequently, in 2022, Hwang et al. published an updated systematic review of TCSW, applying the same inclusion and exclusion criteria as Hajar et al.’s 2015 review [7]. Both systematic reviews found that TCSW was predominantly reported on the faces of women and associated with prolonged use and higher potency of steroids [6,7]. Currently, there are no diagnostic criteria for TCSW, nor are there definitive treatment guidelines for managing this condition. This has resulted in many cases of TCSW being self-diagnosed and lacking confirmation by a clinician [8]. Consequently, debate continues about whether TCSW is a genuine withdrawal phenomenon or an adverse effect of TCS misuse [7]. Due to this uncertainty, in this review, the systematic reviews by Hajar et al. and Hwang et al. were updated, and the existing body of research was further investigated.

## Review

Methodology

A search of Ovid MEDLINE, PubMed, and the Cochrane Library was conducted from October 2020 to June 2024. The search strategy was based on that developed by Hajar et al. [6], which was also utilized by Hwang et al. [7] in their updated review. The search included terms such as TCS withdrawal, addiction, abuse, tolerance, rebound, dependence, rosacea, perioral dermatitis, acneiform eruptions, and rosacea-like eruptions, as outlined in Hajar et al.’s search strategy [6]. All retrieved titles and abstracts were manually screened by a reviewer (H.C.).

Inclusion Criteria

Only English-language articles were considered for inclusion. To qualify for inclusion, articles needed to have described at least one case of steroid withdrawal, defined by the following criteria: (A) a cutaneous eruption that occurred after the use of TCS, either appearing after discontinuation or necessitating elevated doses and applications to prevent its appearance; and (B) The eruption was localized to the site(s) of TCS application. While resolution of the eruption after TCS cessation was considered contributory, it was not required for diagnosis. Studies that defined steroid-induced dermatological conditions (e.g., steroid-induced rosacea (SIR), steroid-induced dermatitis) as “withdrawal” or “rebound” were also included in the review. As these conditions may represent distinct entities, their inclusion introduces clinical heterogeneity. Due to the limited number of available studies, all study designs were included.

Exclusion Criteria

Studies were excluded if there was an absence of a clear temporal relationship between TCS use and the TCS withdrawal eruption, insufficient case descriptions, or a lack of precise data, including exact numbers of patients and documentation of withdrawal symptoms.

Data Extraction

Data from the included studies were extracted by a single reviewer using an Excel spreadsheet. Primary outcomes were clinical features of TCSW, including (A) patient factors, including age, gender, and indication for TCS; (B) TCS factors, including potency and duration of use; (C) signs, including morphology and location; (D) symptoms; and (E) time to onset. Secondary outcomes were treatment modalities for management of TCSW, duration of treatment for TCSW, response to the treatments, and alternative nomenclature used for TCSW.

Quality Assessment

The level of evidence and risk of bias were assessed for each individual study. We used the Oxford Centre for Evidence-Based Medicine’s tool to determine the level of evidence [9,10]. For risk of bias assessment, the Joanna Briggs Institute (JBI) critical appraisal tool was applied across all study designs, including case reports, case series, and cross-sectional studies [11,12]. In accordance with the guidelines of these tools, no quantitative score for risk of bias was assigned. This approach acknowledges that certain items in the risk of bias assessment may carry more weight or may not be applicable, rendering a quantitative score potentially unreliable.

Results

**Figure 1:**
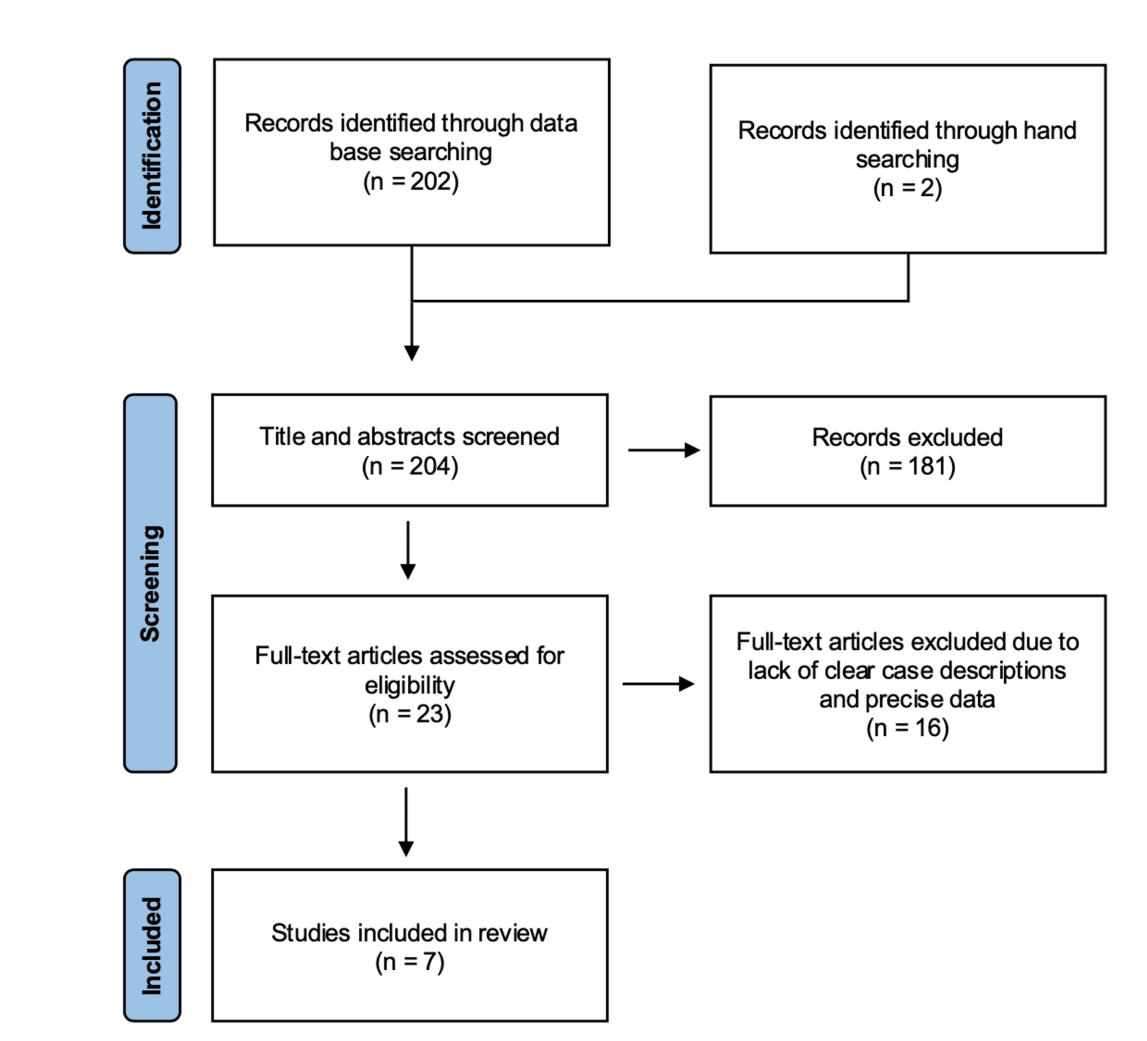
Preferred Reporting Items for Systematic Reviews and Meta-Analyses (PRISMA) flow diagram.

The search yielded a total of 204 articles, which were reviewed and screened down to seven studies (Figure 1). Among these, five were case reports, one was a case series, and one was a qualitative cross-sectional survey based on an online survey. The majority of participants were derived from the patient-reported survey by Barta et al. (n = 1,603), whereas the remaining data were from clinician-reported case reports and a case series. The level of evidence for all included studies was level 4 (Table 1). The risk of bias was relatively consistent across the studies, except for Xu et al. [13] and Barta et al. [8], which exhibited a higher risk than the others (Table 2-4).

**Table 1:** Studies included in the review.

Author	Year	Study type	Level of evidence
Barta et al., 2023 [8]	2023	Qualitative cross-sectional survey	4
Xu et al., 2023 [13]	2023	Case series	4
Katoch et al., 2022 [14]	2022	Case report	4
Feschuk et al., 2023 [15]	2023	Case report	4
Konus et al., 2022 [16]	2022	Case report	4
Li et al., 2022 [17]	2022	Case report	4
Sher et al., 2024 [18]	2024	Case report	4

**Table 2:** Risk of bias for case reports. ^a^: Appraisal questions for case reports: 1. Were the patient’s demographic characteristics clearly described? 2. Was the patient’s history clearly described and presented as a timeline? 3. Was the current clinical condition of the patient on presentation clearly described? 4. Were diagnostic tests or assessment methods and the results clearly described? 5. Was the intervention(s) or treatment procedure(s) clearly described? 6. Was the post-intervention clinical condition clearly described? 7. Were adverse events (harms) or unanticipated events identified and described? 8. Does the case report provide takeaway lessons?

Appraisal questions for case reports^a^								
	1	2	3	4	5	6	7	8
Katoch et al., 2022 [14]	Yes	Yes	Yes	N/A	Yes	Yes	No	Yes
Konus et al., [16]	Yes	Yes	Yes	N/A	Yes	Yes	No	Yes
Li et al., 2022 [17]	Yes	No	Yes	N/A	Yes	Yes	No	Yes
Feschuk et al., 2023 [15]	Yes	Yes	Yes	N/A	Yes	Yes	Yes	Yes
Sher et al., 2024 [18]	Yes	Yes	Yes	N/A	No	Yes	No	Yes

**Table 3:** Risk of bias for case series. ^a^: Appraisal questions for case series: 1. Were there clear criteria for inclusion in the case series? 2. Was the condition measured in a standard, reliable way for all participants included in the case series? 3. Were valid methods used for the identification of the condition for all participants included in the case series? 4. Did the case series have consecutive inclusion of participants? 5. Did the case series have complete inclusion of participants? 6. Was there clear reporting of the demographics of the participants in the study? 7. Was there clear reporting of clinical information of the participants? 8. Were the outcomes or follow-up results of cases clearly reported? 9. Was there clear reporting of the presenting site(s)/clinic(s) demographic information? 10. Was statistical analysis appropriate?

Appraisal questions for case series^a^										
	1	2	3	4	5	6	7	8	9	10	
Xu et al., 2023 [13]	Yes	Yes	Yes	No	No	Yes	No	Yes	Yes	N/A	

**Table 4:** Risk of bias for cross-sectional studies. ^a^: Appraisal questions for cross-sectional studies: 1. Were the criteria for inclusion in the sample clearly defined? 2. Were the study subjects and the setting described in detail? 3. Was the exposure measured in a valid and reliable way? 4. Were objective, standard criteria used for measurement of the condition? 5. Were confounding factors identified? 6. Were strategies to deal with confounding factors stated? 7. Were the outcomes measured in a valid and reliable way? 8. Was an appropriate statistical analysis used?

Appraisal questions for cross-sectional studies^a^								
	1	2	3	4	5	6	7	8
Barta et al., 2023 [8]	No	Yes	No	No	No	No	No	No

Primary Outcomes

The patient features and steroid characteristics are summarized in Table 5. Patient characteristics were derived from both clinician-reported cases and the patient-reported survey, with larger sample sizes primarily reflecting the survey data. The majority of patients with manifestations of TCSW were women (90%, n = 9/10) in clinician-reported cases and adults (92.7%, n = 1,495/1,613) in the pooled dataset, with the latter largely driven by the survey data. The primary indication for TCS use was atopic dermatitis (99.5%, n = 1,605/1,613), primarily derived from the patient-reported survey by Barta et al. [8]. The location of TCS use was mainly on the face (90%, n = 9/10), with the majority using high-potency TCS (80%, n = 4/5), followed by mid-potency TCS (20%, n = 1/5). Data regarding the duration of use was limited, but all analyzed patients (100%, n = 5/5) had been using TCS for more than 12 months.

**Table 5:** Patient and steroid characteristics. ^a^: In total, 1,613 participants were included in the analysis: 1,603 from Barta et al. [8]; 4 from Xu et al. [13]; 2 from Feschuk et al. [15]; and 1 each from Katoch et al. [14], Konus et al. [16], Li et al. [17], and Sher et al. [18]. ^b^: Barta et al. [8] reported that 1,889 adult participants and 271 caregivers of children completed the survey. Among all participants (adults and caregivers combined), 85% were female, and 55% of the caregivers’ children were male. These percentages are based on the total number of participants, not on the patient subset who experienced TCSW; therefore, they are not included. ^c^: Barta et al. [8] reported that among all participants, 73% of adults and 48% of children applied prescription-only TCS to their face. Additionally, among participants who applied prescription-only TCS to their face or genitals, 90% of adults and 54% of children reported symptoms consistent with TCSW. The exact numbers of these patients were not identified; therefore, they are not included. ^d^: Katoch et al. [14] and Xu et al. [13] did not specify the potency of TCS used by the patients. Barta et al. [8] reported that many adults and children used superpotent or potent TCS, but exact numbers are unknown. Additionally, Barta et al. [8] noted that 54% of adults and 36% of children reported using both superpotent and potent TCS. However, these percentages are based on the total number of participants, not on the patient subset who experienced TCSW; therefore, they are not included. ^e^: Barta et al. [8] reported that among a subgroup of 1,702 participants who used prescription-only TCS, 53%, 72%, 75%, and 86% of patients reported symptoms consistent with TCSW after <1, 1-2, 3-5, and ≥6 years of prescription-only TCS use, respectively. Katoch et al. [14] described the duration as “many months,” while Xu et al. [13] did not specify the duration of TCS use. Therefore, these numbers are not included. TCS: topical corticosteroid; TCSW: topical corticosteroid withdrawal

Feature	Number of patients (%)
Age, years	n = 1,613^a^
<18	118 (7.3)
>18	1,495 (92.7)
Gender	n = 10^b^
Female	9 (90)
Indication of TCS use	n = 1,613^a^
Atopic dermatitis	1,605 (99.5)
Rosacea and rosacea-like eruptions	6 (0.3)
Seborrheic dermatitis	2 (0.1)
Location of TCS use	n = 10^c^
Face	9 (90)
Scalp	1 (10)
Potency	n = 5^d^
High	4 (80)
Mid	1 (20)
Duration	n = 5^e^
>12 months	5 (100)

The most common symptoms were burning (89.8%, n = 1,448/1,613), itching (85.6%, n = 1,381/1,613), and skin hypersensitivity (82.3%, n = 1,328/1,613). Major signs included erythema (89.6%, n = 1,446/1,613), desquamation (88.7%, n = 1,431/1,613), oozing (82.5%, n = 1,330/1,613), elephant wrinkles (78.5%, n = 1,266/1,613), and red sleeve (74.1%, n = 1,196/1,613) (Table 6). These proportions were predominantly derived from the patient-reported survey dataset. Elephant wrinkles and red sleeve are unique signs included in the TCSW diagnostic criteria suggested by Sheary [19], which are recognized by many TCSW support organizations, clinicians, and members [7]. Elephant wrinkles refer to apparent thickened skin with reduced elasticity on the anterior knees or extensor elbows [19]. The red sleeve is a rebound eruption on either the upper or lower limb, ending abruptly at the margin of the dorsal and palmar border [19]. Different subtypes, papulopustular and erythroedematous, were identified by Hajar et al. [6]. However, further categorization into subtypes was not performed in our study due to insufficient data. Data regarding the onset of TCSW after TCS cessation were limited, but the two analyzed patients experienced TCSW 4-14 days after stopping TCS (Table 6).

**Table 6:** Clinical features of TCSW. ^a^: Number of patients with reported data for the specified feature. If imprecise data were reported, we estimated the data to the best of our understanding. ^b^: Out of all the studies, only Feschuk et al. [15] clearly specified the onset of TCSW after TCS discontinuation. ^c^: In total, 1,613 participants were included in the analysis: 1,603 from Barta et al. [8]; 4 from Xu et al. [13]; 2 from Feschuk et al. [15]; and 1 each from Katoch et al. [14], Konus et al. [16], Li et al. [17], and Sher et al. [18]. ^d^: Barta et al. [8] defined it as a new hypersensitivity of the skin to water, movement, moisturizer, fabrics, temperature, sunlight, etc. ^e^: Dryness, anhidrosis, madarosis, macules, melasma. TCS: topical corticosteroid; TCSW: topical corticosteroid withdrawal

Feature^a^	Number of patients (%)
Onset of symptoms upon withdrawal	n = 2^b^
4–14 days	2 (100)
Symptoms	n = 1,613^c^
Burning/Stinging	1,448 (89.8)
Pruritis/Itching	1,381 (85.6)
Skin hypersensitivity^d^	1,328 (82.3)
Emotional distress/Depression/Anxiety	1,271 (78.8)
Sleep disturbances	1,260 (78.1)
Fatigue	1,236 (76.6)
Altered thermoregulation (feeling too cold or too hot)	1,214 (75.3)
Eye dryness and irritation	1,075 (66.6)
Nerve pain	1,001 (62.1)
Appetite changes	896 (55.5)
Suicidal ideation	711 (44.1)
Signs	n = 1,613^c^
Erythema/Red skin/Skin flushing	1,446 (89.6)
Desquamation/Peeling/Flaking	1,431 (88.7)
Vesicle/Oozing and weeping skin	1,330 (82.5)
Elephant wrinkles	1,266 (78.5)
Red sleeve	1,196 (74.1)
Swelling/Edema	1,026 (63.6)
Hair loss	948 (58.8)
Enlarged lymph nodes	910 (56.4)
Other^e^	5 (0.3)
Papules	4 (0.2)
Paresthesia	2 (0.1)
Telangiectasia	2 (0.1)

Secondary Outcomes

Treatment data were derived exclusively from clinician-reported cases (n = 10), as treatment information was not available in the survey dataset. Among these cases, the most common strategy was discontinuation of TCS use (50%, n = 5/10), often in combination with other treatments (Table 7). In a case series by Xu et al., four patients were treated with oral abrocitinib tablets (100 mg daily), resulting in resolution for three patients, with one lost to follow-up [13]. These three patients also used topical azelaic acid gel alongside abrocitinib, although the time to resolution varied among them [13]. Additionally, a case report by Katoch et al. stated that oral doxycycline, antihistamines, and topical pimecrolimus resolved papular eruptions but did not address the erythema and post-inflammatory hyperpigmentation [14]. Further treatment with micro botulinum toxin injections was initiated for that patient, leading to a marked reduction in facial erythema and improvement in the red face [14].

**Table 7:** Treatment of TCSW. ^a^: Number of patients with reported data for the specified feature. If imprecise data were reported, we estimated the data to the best of our understanding. ^b^: A total of 10 participants’ treatments were included in the analysis: 4 from Xu et al. [13], 2 from Feschuk et al. [15], and 1 each from Katoch et al. [14], Konus et al. [16], Li et al. [17], and Sher et al. [18]. Treatment data were not reported in Barta et al. [8]. ^c^: Azelaic acid gel was used adjunctively with oral abrocitinib treatment. ^d^: Katoch et al. [14] stated that oral doxycycline, antihistamines, and topical pimecrolimus resolved papular eruptions, but the patient still had a persistent red face and post-inflammatory hyperpigmentation. Therefore, micro botulinum toxin injection was added, resulting in a marked reduction in facial erythema and improvement in the red face. ^e^: One patient from Xu et al. [13] was lost to follow-up due to personal reasons and is therefore not included in the table. TCS: topical corticosteroid; TCSW: topical corticosteroid withdrawal

Feature^a^	Number of patients (%)
Treatment	n = 10^b^
Discontinue TCS use	5 (50)
Tofacitinib and metronidazole gel	1 (10)
Platelet-rich plasma treatment	1 (10)
Oral sarecycline and ruxolitinib cream	1 (10)
Abrocitinib, oral	4 (40)
Azelaic acid gel, topical^c^	3 (30)
Oral doxycycline, antihistamines, and topical pimecrolimus^d^	1 (10)
Micro botulinum toxin injection^d^	1 (10)
Duration and response to treatment	n = 9^e^
0–14 days	0 (0)
2–4 weeks	1 (11.1)
1–3 months	3 (33.3)
≥3–6 months	1 (11.1)
≥6–12 months	1 (11.1)
>1 year	3 (33.3)

The duration and response to treatment varied depending on the treatment modalities used. Two patients from Feschuk et al. discontinued TCS without starting any pharmacological treatment, taking approximately two years to resolve the TCSW manifestations [15]. In contrast, two patients from Xu et al. reached resolution after taking oral abrocitinib with topical azelaic acid for six weeks and eight weeks, respectively [13].

The following alternative nomenclatures were used to describe TCSW in the seven studies in the review: TSWS, SIR, topical corticosteroid-induced rosacea-like dermatitis, and steroid-induced perioral dermatitis. Studies that used one of these nomenclatures were excluded if the definition did not meet our inclusion criteria.

Discussion

TCSW appears to be a clinical adverse effect predominantly observed in patients who have used high-potency TCS over a prolonged period. TCSW was most commonly seen on the face, with adult women presenting more frequently than men, though the reasons for this demographic pattern remain uncertain. Burning, itching, and skin hypersensitivity are common symptoms, with erythema and desquamation being the most prevalent signs. Additionally, there were high rates of oozing, elephant wrinkles, and the red sleeve sign among patients who experienced TCSW. Data regarding the onset of TCSW after TCS cessation were limited, preventing us from drawing definitive conclusions. The features and characteristics of TCSW identified in this updated review are consistent with the previous findings of Hajar et al. and Hwang et al. [6,7]. Notably, most quantitative findings were driven by patient-reported survey data, whereas treatment outcomes were derived from a small number of clinician-reported cases. These findings should be interpreted with caution, as the pooled estimates are derived from heterogeneous data sources with varying denominators.

This review was unable to identify the most effective management strategy for TCSW. Steroid discontinuation was the primary management approach, but it may not be the best option for patients seeking a quick resolution, as evidenced by the two patients from Feschuk et al. who took approximately two years to resolve their TCSW manifestations after discontinuing TCS [15]. Immune-modulating medications, such as the JAK1 inhibitor abrocitinib, were used in cases of TCS-induced rosacea and resolved symptoms fairly quickly [13]. Xu et al. noted that due to TCSW’s rebound phenomenon involving hormones and the continued release of inflammatory factors, SIR takes a long time to respond to conventional treatments such as topical antibiotics, oral antibiotics, and antihistamines [13]. Therefore, they used the JAK1 inhibitor abrocitinib in their patients [13]. Additionally, platelet-rich plasma treatment has recently gained attention as a treatment strategy for TCSW [16,20].

Due to insufficient data, this review was unable to classify TCSW into different subtypes, such as papulopustular and erythroedematous, as identified by Hajar et al. [6]. It is important to recognize that variations in the underlying etiology of these subtypes may significantly influence treatment approaches. Future randomized studies are needed to better elucidate these differences and guide appropriate management of TCSW.

Furthermore, diagnostic criteria that can effectively differentiate true TCSW from other dermatologic conditions are needed. The majority of patients who reported experiencing TCSW in this review were diagnosed with eczema, raising the possibility that eczema flare-ups were mistakenly thought to be TCSW. In 2015, Hajar et al. suggested criteria to differentiate TCSW from atopic dermatitis flare-ups [6]. They recommended favoring TCSW over a flare-up if: (1) burning is the prominent symptom, (2) erythema occurs after TCS discontinuation, and (3) a history of frequent and prolonged TCS usage on the face or genital region is present [6]. In 2018, Sheary suggested diagnostic criteria for TCSW very similar to those of Hajar et al., with the only difference being that they suggested itch as an essential symptom instead of burning [19]. In this review, both burning and itching were the most common symptoms presented in patients with TCSW, supporting the use of both criteria. However, more data from randomized studies are needed in the future to determine the clinical significance, sensitivity, and specificity of these diagnostic criteria.

Limitations

Our review process has several limitations, including the use of a single reviewer for screening studies and extracting data. Additionally, we included heterogeneous study designs, incorporating all available studies regardless of type or quality due to the limited data on this topic. Notably, the majority of the data was derived from a single qualitative cross-sectional study based on online patient survey responses. These self-reported data are inherently subjective and may introduce selection and recall bias, as clinical features were not independently verified by clinicians [8]. Therefore, these findings may not accurately reflect the real-world presentation of TCSW.

During the selection process, many inconsistencies were found in definitions and diagnostic criteria for the same condition. For example, SIR was defined as withdrawal from TCS use in some studies, while others defined SIR as a side effect of TCS occurring during use [13,16-18,21,22]. This lack of uniformity in definitions and diagnoses of conditions secondary to TCS use made it difficult to select studies for inclusion in this review, further contributing to the potential misrepresentation of TCSW in the clinical arena.

In addition to a lack of diagnostic uniformity and heterogeneous study designs, variability in the clinical conditions represented within this review was also observed. We included studies by Sher et al., Li et al., and Xu et al. that describe related steroid-induced dermatoses, such as SIR, perioral dermatitis, and rosacea-like eruptions, within our analysis of TCSW. These studies were included due to the limited availability of evidence on TCSW in the literature. However, these conditions may represent distinct clinical entities rather than true TCSW. Although they share overlapping features, they may differ in underlying pathophysiology and treatment response. This variability limits direct comparability across studies and may affect the interpretation of pooled clinical features and treatment outcomes, as some findings attributed to TCSW may instead reflect other steroid-induced dermatologic conditions.

The potential for misclassification within TCSW criteria also has important implications for understanding adverse outcomes. Regardless of whether this condition represents a side effect or withdrawal from TCS, it is evident that the risk of adverse outcomes is likely driven by inappropriate use of TCS. According to Barta et al., participants frequently exceeded recommended usage, with many applying it at least once a day for more than 15 days a month [8]. The increasing “steroid phobia” on social media platforms, also evidenced by survey responses indicating that anxiety was the most common new symptom developed by patients with eczema treated with corticosteroids, may lead to medication nonadherence and treatment complications, potentially exacerbating TCSW due to inappropriate TCS use [2-4,8].

## Conclusions

TCSW appears to be a clinical adverse effect that typically occurs after the prolonged use of high-potency TCS, primarily on the face. Reported symptoms commonly include burning, itching, and skin hypersensitivity, while frequent signs are erythema and desquamation. Unique features such as elephant wrinkles and the red sleeve sign were also noted. Findings should be interpreted with caution, given the limited, heterogeneous, and predominantly low-quality nature of the available data, much of which is self-reported patient experiences. Inconsistent diagnostic criteria and limited data availability necessitated the inclusion of related steroid-induced dermatoses within TCSW analyses, which further limits the interpretability of findings. Increasing patient anxiety and the spread of misinformation regarding TCSW on social media may influence perceptions of corticosteroid use and contribute to nonadherence or inappropriate use, further complicating management. There remains a need for higher-quality research, including well-designed cohort studies and randomized controlled trials, to better characterize the potential etiologies, clinical features, and optimal management strategies for TCSW. Establishing standardized diagnostic criteria and clearer definitions to distinguish TCSW from other dermatologic conditions, as well as from adverse effects related to corticosteroid misuse, is essential. Collaborative efforts among dermatologists, researchers, and patient advocacy groups will be important to advance the understanding of TCSW and lead to better patient outcomes.
